# Disintegration Behavior in Industrial Conditions of Poly(lactic acid)/Poly(hydroxybutyrate-*co*-valerate) Blends

**DOI:** 10.1021/acsomega.6c03726

**Published:** 2026-08-04

**Authors:** Vito Gigante, Corrado Sciancalepore, Róbert Várdai, Duccio Gallichi-Nottiani, Nóra Hegyesi, Laura Aliotta, Daniel Milanese, Béla Pukánszky, Andrea Lazzeri

**Affiliations:** † Department of Civil and Industrial Engineering, 9310 University of Pisa, Pisa 56126, Italy; ‡ 9370 National Interuniversity Consortium of Materials Science and Technology (INSTM), Florence 50121, Italy; § Department of Physical Chemistry and Materials Science, Faculty of Chemical Technology and Biotechnology, Budapest University of Technology and Economics, Budapest 1111, Hungary; ∥ Institute of Materials and Environmental Chemistry, Hun-Ren Research Centre for Natural Sciences, Budapest 1117, Hungary; ⊥ Department of Industrial Systems and Technologies, University of Parma, Parma 43121, Italy

## Abstract

This study provides a comprehensive evaluation of the disintegration kinetics of poly­(lactic acid) (PLA)/poly­(hydroxybutyrate-*co*-valerate) (PHBV) blends by exploring the full compositional spectrum (neat polymers and three intermediate ratios), bridging the gap between degradation behavior, the underlying thermal and mechanical properties, and crystallinity. Extrusion and injection molding techniques were used as processing techniques, and they were instrumental in defining the blends’ final properties and microstructural organization. The resulting crystallinity was identified as a primary factor influencing the disintegration behavior, acting in conjunction with the surface-area-to-volume ratio dictated by sample thickness. The results suggest that PHBV negatively affects the fragmentation of the blends, likely due to its inherently high crystallinity and hydrophobicity, potentially assisted by a constrained amorphous fraction that maintains material stiffness near typical industrial composting temperatures. This behavior was consistently observed in both laboratory-scale and pilot-scale disintegration tests. To gain deeper insights into the disintegration mechanisms, scanning electron microscopy was carried out on the surfaces of the degraded samples, revealing morphological changes and degradation patterns correlated with the structural and compositional differences among the materials. Overall, this study highlights the need to correlate morphology and thermal characteristics with processing history and disintegration conditions to accurately assess the disintegration behavior of biodegradable polymer materials.

## Graphical abstract

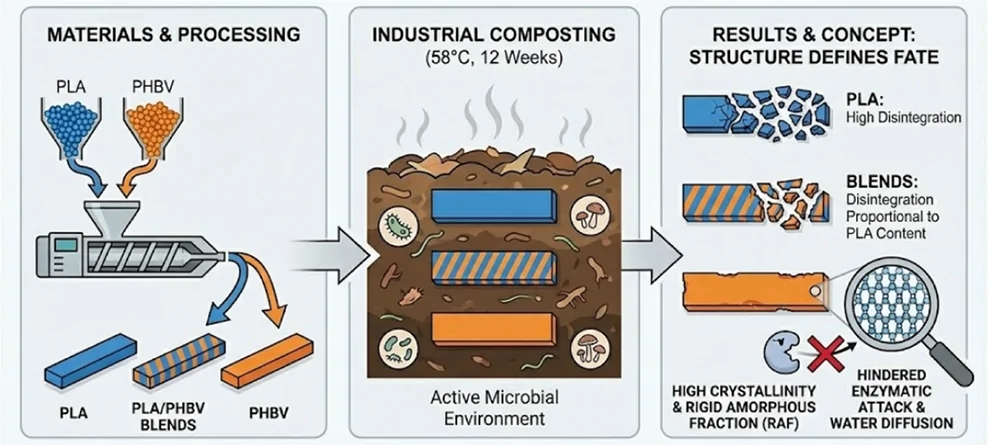

## 1 Introduction

The continuous growth of plastic production represents a critical global challenge. In 2024, global plastic production exceeded 400 million tons, and current projections indicate that this value could triple by 2060 in the absence of effective mitigation policies.^1^ Moreover, more than 220 million tons of plastic waste were generated, with inadequate management leading to significant environmental pollution.^2^ These figures clearly highlight the urgency of developing sustainable alternatives to traditional plastics in order to reduce environmental impact and dependence on fossil-based materials.^3^ Indeed, a significant part of current polymer research is therefore focused on the development of sustainable materials such as bioderived and/or compostable polymers.^4^ In this context, biodegradable and compostable plastics (produced from either renewable biological resources or fossil raw materials) have attracted considerable interest.^5^ However, according to the principles of circular economy and waste hierarchy strategies, these materials should be employed primarily in applications in which reduction, reuse, or recycling are not feasible.^6^ Despite their potential, it is essential to critically assess whether bioplastics consistently meet biodegradability expectations under real disposal conditions. In fact, their degradation behavior can strongly depend on several factors, including processing history, chemical structure, morphology, specific environment in which degradation occurs and the enzymes that form in such environments.^7^

Within this framework, the present work focuses on blends of two of the most widely used biopolymers currently available on an industrial scale: poly­(lactic acid) (PLA), produced via lacto-fermentation of carbohydrates^8^) and poly­(hydroxybutyrate-*co*-valerate) (PHBV), synthesized by the bacterial fermentation of biomass.^9^ The objective is to investigate their disintegration behavior under industrial composting conditions across the full composition range, from neat PLA to neat PHBV) and to correlate the results with their morphological and thermo-mechanical properties.

PLA is characterized by several well-known limitations, including low crystallinity, particularly in thermoformed and injection-molded products,^10^ limited toughness,^11^ and relatively small polarity.^12^ Conversely, PHBV exhibits drawbacks related to its brittleness and challenging processing conditions.

Generally, physical blending represents an effective strategy to overcome such limitations by combining different polymers to tailor properties, reducing costs, and enhancing overall performance.^13^ In PLA/PHBV systems, potential synergistic effects arise from the ease of processing, stiffness, and mechanical strength of PLA combined with the distinct crystallization kinetics and biodegradation behavior of PHBV.^14^ Finely dispersed PHBV crystals may act as both fillers and nucleating agents for PLA,^15^ moreover Mokofeng et al.^16^ demonstrated that PLA/PHBV blends do not only improve the mechanical performance of PHBV but also provide enhanced gas barrier properties. From an economic perspective, blending PHBV with PLA can also reduce costs, as PHBV production remains relatively expensive due to the fermentation and purification processes.^17^

In the context of single-use rigid packaging, simple physical blending, without chemical compatibilizers or reactive extrusion, has been reported to promote biodegradability.^18^ In the absence of compatibilizers, PLA and PHBV remain immiscible, with one polymer forming a dispersed phase within the matrix of the other. Under such conditions, processing parameters play a crucial role in determining the final morphology and macroscopic properties of the blends.^19^ In this regard, Nanda et al.^20^ produced a wide range of PLA/PHBV blends via melt processing and demonstrated that careful process engineering can yield materials with properties comparable to conventional commodity polymers used in rigid packaging applications.^21^

To further advance the use of these systems, a detailed investigation of their disintegration behavior is required under industrial composting conditions. While the disintegration and biodegradation of PLA under simulated composting conditions (58–60 °C) have been extensively documented,^22^ the behavior of PHBV remains more complex. Several studies have reported that PHBV degradation strongly depends on environmental conditions, microbial activity, and material structure.^23,24^ Although PHBV is known to undergo enzyme-catalyzed hydrolysis,^25^ its degradation rate is often governed by crystallinity, lamellar structure, and enzyme accessibility rather than by abiotic hydrolysis alone.^26,27^ Based on these considerations, the present study aims to investigate PLA/PHBV blends systematically across a wide composition range, with the dual objectives of optimizing processing conditions to achieve suitable macroscopic properties and elucidating how crystallinity and morphology influence disintegration behavior under laboratory and pilot-scale composting conditions. Attention is devoted to assessing the role of sample thickness and to evaluating whether, in the absence of tailored enzyme selection, PHBV can effectively disintegrate in a naturally developed microbial environment, potentially facilitating PLA degradation. Conversely, the study also explores whether the enhanced crystallization and crystal orientation of PHBV may instead inhibit disintegration, leading to reduced biodegradation.

## 2 Materials and Methods

### 2.1 Materials

The materials used in this study are listed below.

- Poly­(lactic acid) (PLA), commercial grade PLA Ingeo 3100HP, containing 0.3% of D-lactic acid units [melt flow index (MFR): 24 g/10 min (210 °C, 2.16 kg); nominal average molar mass: 120 000 g/mol; density: 1.24 g/cm^3^. It is a PLA grade designed to crystallize during processing in most conventional injection molding equipment.^28^
- Poly­(hydroxybutyrate-*co*-valerate) (PHBV) commercial grade PHI 002 was supplied by NaturePlast (NPL, Rue François Arago, France). It is a thermoplastic resin containing approximately 5 wt % of 3-hydroxyvalerate units and its density is 1.24 g·cm^–3^.

The compositions of the blends investigated in this work are reported in Table 1.

**Table 1:** Composition and Designation of the PLA/PHBV Blends Investigated in This Study

Blend name	PLA wt %	PHBV wt %
PLA	100	0
PLA75PHBV25	75	25
PLA50PHBV50	50	50
PLA25PHBV75	25	75
PHBV	0	100

### 2.2 Sample Preparation

PLA, PHBV, and their blends were compounded using a semi-industrial twin-screw extruder (Comac EBC 25HT, L/D = 44; Comac, Cerro Maggiore, Italy), operating at a feed rate of 15 kg·h^–1^ and a screw speed of 230 rpm. The temperature profile along the barrel (in °C) was set as follows: 150–175–190–190–195–195–190–190–180–180–170.

This “hump-shaped” temperature profile was selected based on previous processing experiences^29^ with PLA-based systems, as it promotes improved melt mixing in the central, high-temperature zones and enhances pressure buildup toward the die. Prior to extrusion, both polymers were dried at 60 °C using a Piovan DP 604 dryer (Piovan S.p.A., Verona, Italy).

After the compounding process, the pellets were molded into square-shaped specimens (50 × 50 mm with two different thicknesses: 0.5 and 2 mm) using a Demag IntElect 50/330–100 injection molding machine (Sumitomo Demag GmbH, Schwaig, Germany), as shown in Figure 1. The injection molding conditions used for each composition are reported in Table 2.

**Figure 1:**
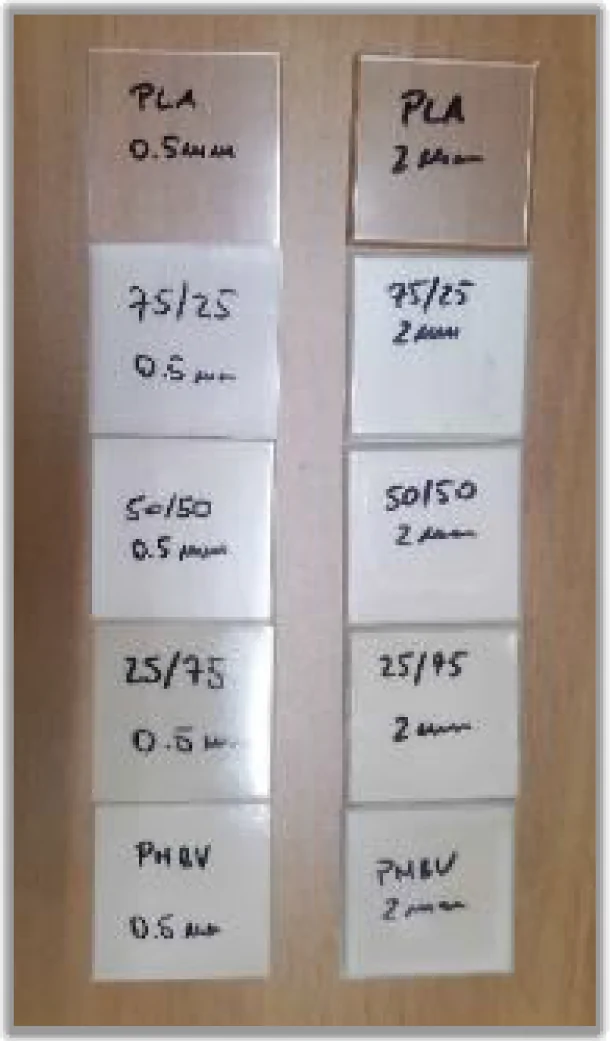
Square specimens (50 × 50 mm) injection-molded with two different thicknesses (0.5 and 2 mm) used for thermal, morphological, and disintegration analyses.

**Table 2:** Injection Molding Conditions of the Blends Produced

**PLA**								
Thickness (mm)	Injection pressure [bar]	Injection speed [mm/s]	Holding time [s]	Holding pressure [bar]	Cooling time [s]	Cushion [mm]	Barrel Temp. [°C]	Mold Temp. [°C]
0.5	1400	80	18	1000	50	2–3	190–185–180–175	40
2	750	80	18	550	60	2–3	190–185–180–175	40
**PLA75PHBV25**								
0.5	1000	80	18	650	50	2	190–185–180–175	40
2	500	80	18	400	60	2–3	190–185–180–175	40
**PLA50PHBV50**								
0.5	900	80	18	620	50	1–2	190–185–180–175	40
2	600	80	18	400	60	2–3	190–185–180–175	40
**PLA25PHBV75**								
0.5	700	80	18	400	50	1–2	190–185–180–175	40
2	500	80	18	300	60	3–4	190–185–180–175	40
**PHBV**								
0.5	500	80	18	225	50	3–4	190–185–180–175	40
2	200	80	18	120	60	3–5	190–185–180–175	40

### 2.3 Characterization

#### 2.3.1 Melt Flow Rate

The melt flow behavior of the extruded granules was evaluated using a Melt Flow Tester M20 (CEAST, Torino, Italy). MFR measurements were performed according to ISO 1133, using a load of 2.16 kg, a test temperature of 190 °C with a preheating without weight of 60 s.

#### 2.3.2 Dynamic-Mechanical-Thermal (DMTA) Analysis

The viscoelastic properties of the samples were investigated by dynamic mechanical thermal analysis (DMTA) using Q800 Dynamic Mechanical Analyzer (TA Instruments, Newcastle, DE, USA). Rectangular specimens (30 × 10 × 2 mm) were tested in single-cantilever mode. The oscillation amplitude was set to 10 μm at a frequency of 1 Hz. The temperature was ramped from −60 to 180 °C at a heating rate of 3 °C·min^–1^, following an initial equilibration time of 15 min. The storage modulus (*E′*), loss modulus (*E″*), and damping factor (tan *δ = E″/E*′) were recorded as functions of temperature.

#### 2.3.3 Fourier Transform Infrared (FTIR) Spectroscopy

Fourier transform infrared (FTIR) spectroscopy was carried out using a Spectrum Two spectrophotometer (PerkinElmer, Waltham, MA, USA) equipped with an attenuated total reflectance (ATR) accessory. Spectra were collected in the wavenumber range of 400–4000 cm^–1^, with a resolution of 4 cm^–1^ and 16 scans per measurement. FTIR analyses were performed on samples before and after the disintegration tests to identify possible changes in chemical structure induced by degradation.

#### 2.3.4 Scanning Electron Microscopy (SEM)

Scanning electron microscopy (SEM) was employed to investigate surface morphology and microstructural features of the samples before and after the disintegration tests. Surfaces were observed using a field-emission scanning electron microscope (Nova NanoSEM 450 FEG, Thermo Fisher Scientific, Hillsboro, OR, USA) at various magnifications. Cross-sectional morphologies were examined using an FEI Quanta 450 FEG microscope (Thermo Fisher Scientific), operated at an accelerating voltage of 3 kV and a beam current of approximately 50 pA. Prior to observation, cryo-fractured surfaces were sputter-coated with a thin platinum layer (∼8 nm) to minimize charging effects.

#### 2.3.5 Differential Scanning Calorimetry Analysis

Differential scanning calorimetry (DSC) analyses were performed using a TA Instruments Q200 calorimeter (TA Instruments, Newcastle, DE, USA). Nitrogen was used as purge gas at a flow rate of 50 mL·min^–1^. Temperature and enthalpy calibrations were carried out using indium as a standard. To correlate thermal properties with disintegration behavior, only the first heating scan was considered, as it reflects the thermal history imposed during injection molding. Samples were taken consistently from the same region of each specimen to minimize variations associated with nonuniform cooling across the thickness. Specimens, weighing between 11 and 15 mg, were sealed in aluminum pans. Samples were rapidly cooled from room temperature to −50 °C and held for 1 min, then heated to 200 °C at a rate of 10 °C·min^–1^. Glass transition temperature (*T*_g_), cold crystallization temperature (*T*_*cc*_), and melting temperature (*T*_*m*_) were determined from the thermograms. When applicable, the degree of crystallinity (*X*_*cc*_) of PLA and PHBV was calculated according to eq 1:

1 $$X_{CC}=\frac{\Delta H_{m,PLA\left(or\;PHBV\right)}-\Delta H_{cc,PLA\left(or\;PHBV\right)}}{{{{\Delta H}_{m}}^{0}}_{PLA\left(or\;PHBV\right)}\times wt\%\;of\;PLA\left(or\;PHBV\right)}$$

where Δ*H*_*m*_ and Δ*H*_*cc*_ represent the melting and cold crystallization enthalpies, respectively, and Δ*H* ^0^_m_ is the theoretical melting enthalpy of the fully crystalline polymer (93 J·g^–1^ for PLA^30^ and 143 J·g^–1^ for PHBV^31^).

#### 2.3.6 Lab-Scale Disintegration Test

Disintegration tests were carried out under laboratory composting conditions according to the ISO 20200:2015 standard, *Plastics—Determination of the degree of disintegration of plastic materials under simulated composting conditions in a laboratory-scale test*.^32^ For each composition, square specimens with lateral dimensions of 25 × 25 mm and thicknesses of 0.5 and 2 mm were prepared. Polypropylene containers were used as composting reactors. In accordance with the standard, each reactor was equipped with a 5 mm side hole to ensure adequate air circulation. The composition of the dry synthetic solid waste used as composting medium is reported in Table 3. The mixture was hydrated with deionized water to achieve a moisture content of 55 wt %.

**Table 3:** Composition of the Dried Synthetic Solid Waste

Component	wt %	Supplier
Sawdust	40	OBI, Italy
Rabbit feed	30	Vitakraft, Germany
Ripe compost	10	Punta allo Zero S.r.l. for Coop, Italy
Corn starch	10	Unilever, Italy
Sugar	5	Maxi S.r.l., Italy
Corn seed oil	4	Oleificio Salvadori S.r.l., Italy
Urea	1	Merck-Sigma, Germany

Prior to burial, test specimens were dried at 40 °C to constant mass, individually weighed, and then embedded into the composting medium. To maintain a test material-to-compost ratio of 0.5 wt %, the number of samples per reactor depended on specimen thickness: one 2 mm-thick specimen was sufficient, whereas four 0.5 mm-thick specimens of the same composition were placed in each reactor to achieve the same ratio. It is worth noting that while this approach strictly adheres to the standard mass-based requirements, it intrinsically provides a significantly larger exposed surface area for the thinner specimens, which inevitably influences the kinetics of the surface-mediated enzymatic degradation. The specimens were placed onto a stainless-steel grid with a mesh size of 2 mm. The total mass of each reactor was recorded before placing the reactors in a ventilated oven (ISCO NSV 9090) maintained at 58 ± 2 °C. Reactors were periodically weighed throughout the test, manually mixed, and rehydrated in accordance with the standard protocol. The total test duration was 88 days. To monitor disintegration, specimens were recovered weekly, gently rinsed with deionized water to remove fragments smaller than 2 mm, dried overnight at 40 °C, and weighed to determine mass loss. Optical microscopy images were acquired at each sampling interval. The degree of disintegration (*D*) was calculated as the mean value of three specimens. The dry mass (DM) of the compost was determined before and after the test by drying samples of known volume at 105 °C to constant mass. The dried compost was subsequently calcined at 550 °C for 8 h (LKN 75, Nannetti), cooled overnight, and weighed. This procedure was repeated until constant mass was achieved. The volatile solids (VS) content was calculated as the mass loss during calcination relative to DM. The variation in volatile solids content (*R*) before and after the test was calculated with eq 2:

2 $$R=\frac{\left[M_{i}\times {\left(DM\right)}_{i}\times {\left(VS\right)}_{i}\right]-\left[M_{f}\times {\left(DM\right)}_{f}\times {\left(VS\right)}_{f}\right]}{\left[M_{i}\times {\left(DM\right)}_{i}\times {\left(VS\right)}_{i}\right]}\times 100$$

where *M* is the mass of the synthetic waste, *DM* is the dry mass, and VS is the volatile solids content; subscripts *i* and *f* refer to initial and final values, respectively. According to ISO 20200, the test is considered valid if *R* ≥ 30%.

#### 2.3.7 Disintegration Test under Pilot-Scale Conditions

To validate the laboratory-scale findings under more realistic industrial conditions, pilot-scale disintegration tests were performed exclusively on PHBV, which exhibited the lowest disintegration rate among all investigated materials and to confirm the results. Two sets of PHBV specimens were tested: freshly injection-molded samples and samples aged for six months after molding, in order to evaluate the potential influence of aging on disintegration behavior.

Pilot-scale disintegration tests were conducted by a certified testing facility (ARCHA S.r.l., Pisa, Italy) in accordance with the ISO 16929:2021 standard (*Plastics—Determination of the degree of disintegration of plastic materials under defined composting conditions in a pilot-scale test*). Compliance with disintegration requirements (>90% within 12 weeks), as defined in EN 13432, was determined using the quantitative sieving method.

For PHBV, the composting inoculum was prepared using the following composition: fruit and vegetable waste (50 wt %, consisting of 12% banana, 28% apple, 12% pear, 12% carrot, 24% potato, and 12% leek), mature compost (5 wt %), rabbit feed pellets (30 wt %), and bulking material (wood bark, 15 wt %). Compost and bark were sieved to 5 cm prior to mixing, while fruits and vegetables were cut into pieces smaller than 5 cm. The mature compost was stored at 4 ± 1 °C for a maximum period of one month prior to use. Three composting vessels were employed: two control reactors containing inoculum only and one reactor containing the test material. Each vessel was filled with 15 kg of inoculum. PHBV specimens were added at a concentration of 10 g·kg^–1^ of compost, using square samples with the final dimensions of 50 × 50 mm. The inoculum and test material were thoroughly mixed prior to sealing the vessels, which were then placed into an incubation chamber at a controlled temperature. Throughout the test, compost maturity was verified, oxygen concentration in the exhaust air was maintained above 10%, and pH values remained above 5, in accordance with ISO 16929 requirements. Disintegration results were expressed as the percentage of material disintegration after 12 weeks; in this manner, the extent of fragmentation was quantified as the fraction of the initial dry mass that fully disintegrated under pilot-scale composting conditions.

## 3 Results and Discussion

### 3.1 Melt Flow Rate (MFR)

The melt flow rate (MFR) represents a key parameter for evaluating the processability of polymeric materials, as it reflects their ability to flow under specific temperature and load conditions. In the case of PLA/PHBV blends, the measured MFR values fall between those of the neat components. As shown in Figure 2, neat PLA has an MFR of 2.3 g × 10 min^–1^, whereas PHBV displays the significantly larger value of 10.1 g × 10 min^–1^. The blends have intermediate MFR values, following an approximately linear trend as a function of composition. This quasi-linear behavior can be attributed to the additive nature of the rheological contributions of the two polymers, despite their immiscibility.^33^ Since MFR is inversely proportional to melt viscosity, the measured values reflect the combined influence of the viscosities of PLA and PHBV;^34^ indeed when a high-viscosity polymer, such as PLA, is blended with a lower-viscosity polymer, like PHBV, the MFR of the blend is expected to fall between the values of the two pure components.

**Figure 2:**
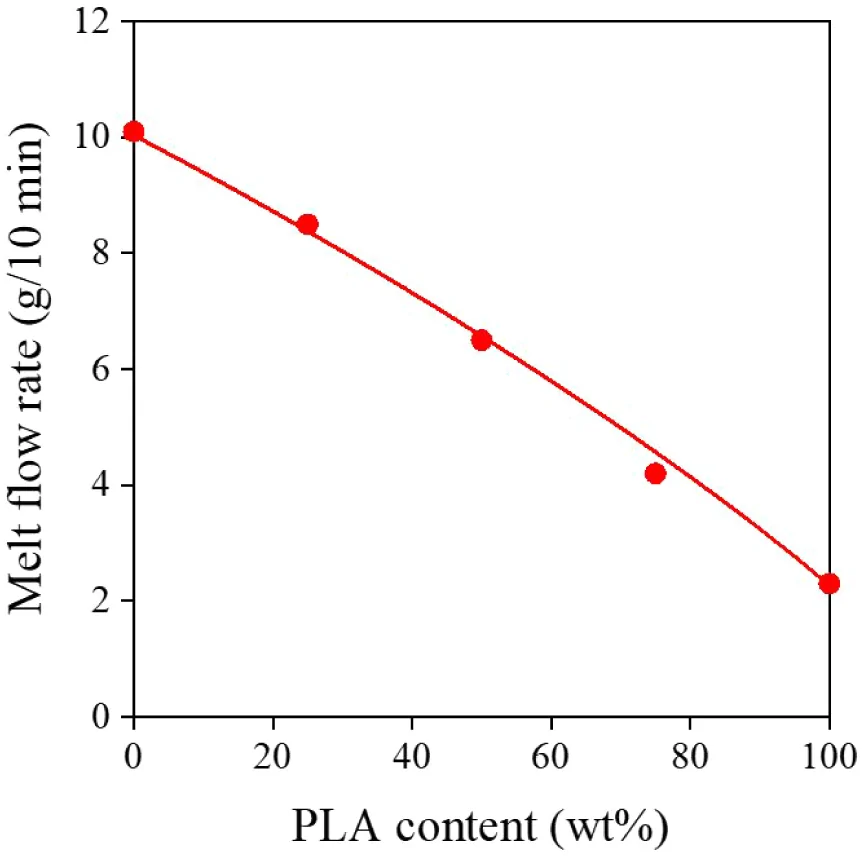
Melt flow rate (MFR) of extruded PLA/PHBV blend granules as a function of composition.

The observed linear dependence suggests that the overall flow behavior of the blends can be reasonably described as a weighted average of the rheological responses of the two phases. Moreover, the absence of strong interactions or pronounced synergistic or antagonistic effects indicates that melt flow is primarily governed by the intrinsic viscosities of PLA and PHBV rather than by complex interfacial dynamics. Although phase separation is expected in this immiscible system, it does not significantly disrupt melt flow, as evidenced by the lack of extremes in MFR, which have been reported for other polymer blend systems.^35^

### 3.2 Morphological Analysis of the Cryogenically Fractured Sections

The morphology of the PLA/PHBV blends was investigated by scanning electron microscopy (SEM) on cryogenically fractured surfaces, as shown in Figure 3. Neat PLA exhibits a homogeneous and featureless morphology, characteristic of the fracture surface of an amorphous polymer.

**Figure 3:**
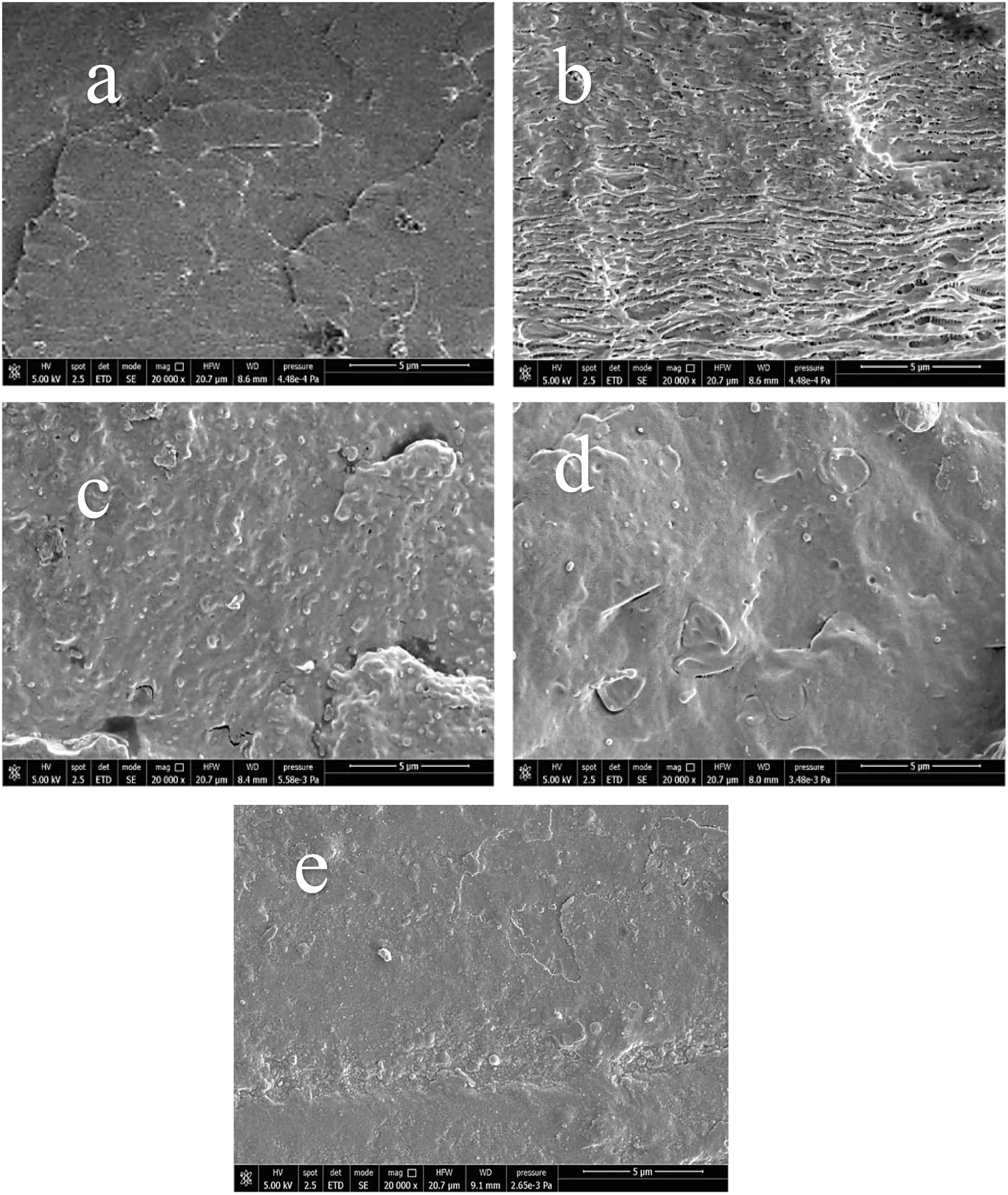
SEM micrographs (20,000× magnification) of cryogenically fractured surfaces of (a) neat PLA, (b) PLA75PHBV25, (c) PLA50PHBV50, (d) PLA25PHBV75, and (e) neat PHBV.

At a PHBV content of 25 wt % (PLA75PHBV25), the dispersed phase can be clearly distinguished. To quantitatively assess the morphology, a domain size distribution analysis was performed on the SEM micrographs (Figure 4).

**Figure 4:**
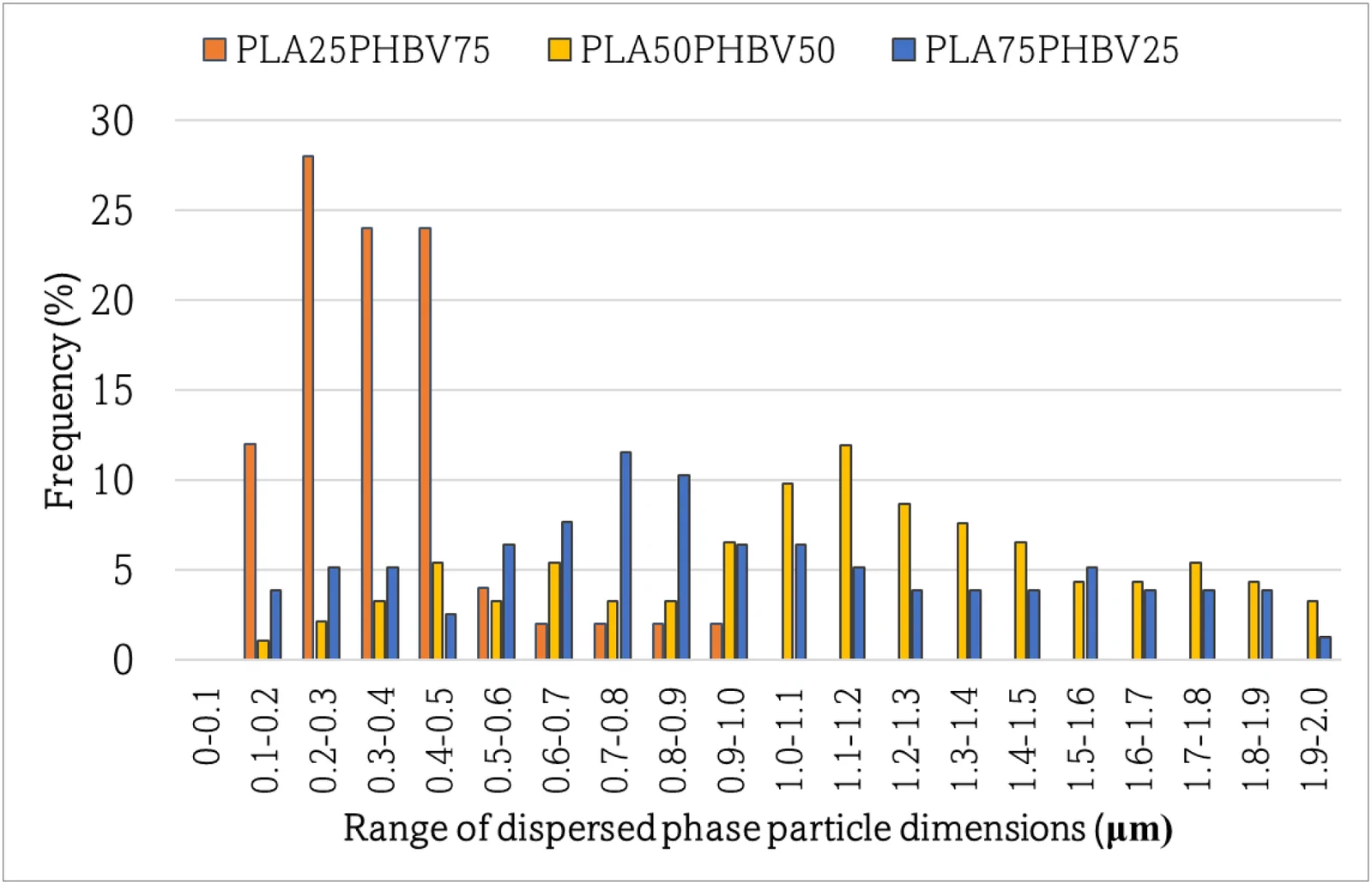
Domain size distribution of the dispersed phase for the intermediate PLA/PHBV blends. Data were obtained through quantitative image analysis of the cryogenically fractured surfaces observed via SEM.

For this composition, the PHBV domains dispersed within the PLA matrix possess a size distribution predominantly in the submicrometric range, with a peak frequency between 0.7 and 0.9 μm. This morphology and domain size are consistent with the typical phase separation behavior of conventional, uncompatibilized immiscible polymer blends.^15^ As the PHBV fraction increases to 50 wt % (PLA50PHBV50), the system exhibits the expected morphological evolution for melt-compounded immiscible blends: the domain size distribution broadens significantly, with particle dimensions extending up to and beyond 2.0 μm. This broader distribution reflects pronounced domain coalescence, marking the onset of cocontinuity and phase inversion.^36^ Finally, in blends containing 75 wt % PHBV (PLA25PHBV75), the phase morphology is fully inverted. In this composition, fine PLA domains are dispersed within the continuous PHBV matrix, displaying a highly concentrated and narrow size distribution that peaks sharply between 0.2 and 0.3 μm. This quantitative morphological assessment confirms the typical lack of strong interfacial interaction in plain PLA/PHBV systems, which is a critical baseline for interpreting the surface-driven disintegration kinetics discussed in the following sections.

When one phase is dispersed within the other, SEM micrographs commonly reveal domain sizes in the submicron to low-micron range (approximately 0.7–1.2 μm), often with a bimodal size distribution reflecting the coexistence of small droplets and larger domains.^37^ The morphologies observed in the present work are fully consistent with the expected behavior of PLA/PHBV systems processed by melt compounding. This morphological consistency is particularly relevant in view of the subsequent discussion of thermal properties and disintegration behavior.

### 3.3 Thermal Properties: Differential Scanning Calorimetry (DSC)

The DSC results obtained from the first heating scan for both specimen thicknesses are reported in Table 4 and Figure 5. The analysis focuses exclusively on the first heating run, as it reflects the thermal history imposed during injection molding and therefore represents the relevant approach to correlate thermal properties with disintegration behavior. Therefore, it represents the relevant approach to correlate the material’s initial structure, specifically its crystallinity and dispersed phase morphology, with its subsequent disintegration behavior. In neat PLA, the thermogram exhibits a glass transition (*T*_g_) in the range of 55–65 °C, identified by a characteristic baseline shift. Immediately above *T*_g_, a small endothermic peak is observed.^38^ This phenomenon arises from the relaxation of polymer chains trapped in a nonequilibrium state during cooling, resulting in an enthalpy recovery upon heating.^39^

**Table 4:** Differential Scanning Calorimetry (DSC) Data Obtained from the First Heating Scan of PLA/PHBV Specimens, Including Glass Transition Temperature (*T*_g_), Cold Crystallization Temperature (*T*_cc_), Melting Temperature (*T*_m_), and Associated Enthalpy Values

Blend name (0.5 mm)	*T*_g,PLA_ (°C)	PHBV, RAF related transition (°C)	*T*_cc,PLA_ (°C)	*T*_m,PLA_ (°C)	*T*_m,PHBV_ (°C)	ΔH_m,TOTAL_ (J/g)	*X*_cc,PLA_	*X*_cc,PHBV_
*PLA*	61.4	-	95 8	175.0	-	-	25.7	-
*PLA75PHBV25*	51.4	^a^	87.7	174.8^b^		33.2		
*PLA50PHBV50*	54.5	^a^	91.2	178.8^b^		29.7		
*PLA25PHBV75*	55.6	^a^	88.0	176.9^b^		16.5		
*PHBV*	-	61.1	-	-	177.3	-	-	65.7
Blend name (2 mm)	*T*_g,PLA_ (°C)	PHBV, RAF related transition (°C)	*T*_cc,PLA_ (°C)	*T*_m,PLA_ (°C)	*T*_m,PHBV_ (°C)	Δ*H*_m,TOTAL_ (J/g)	*X*_cc,PLA_	*X*_ccPHBV_
*PLA*	61.2	-	96.1	178.0	-	-	19	-
*PLA75PHBV25*	52.8	^a^	85.2	175.2^b^		30.9		
*PLA50PHBV50*	53.5	^a^	91.0	178.3^b^		23.1		
*PLA25PHBV75*	59.0	^a^	89.2	177.1^b^		15.0		
*PHBV*	-	61.1	-	-	176.0	-	-	67.8

a Not visible.

b The melting temperatures of the neat polymers are so close that they cannot be distinguished.

**Figure 5:**
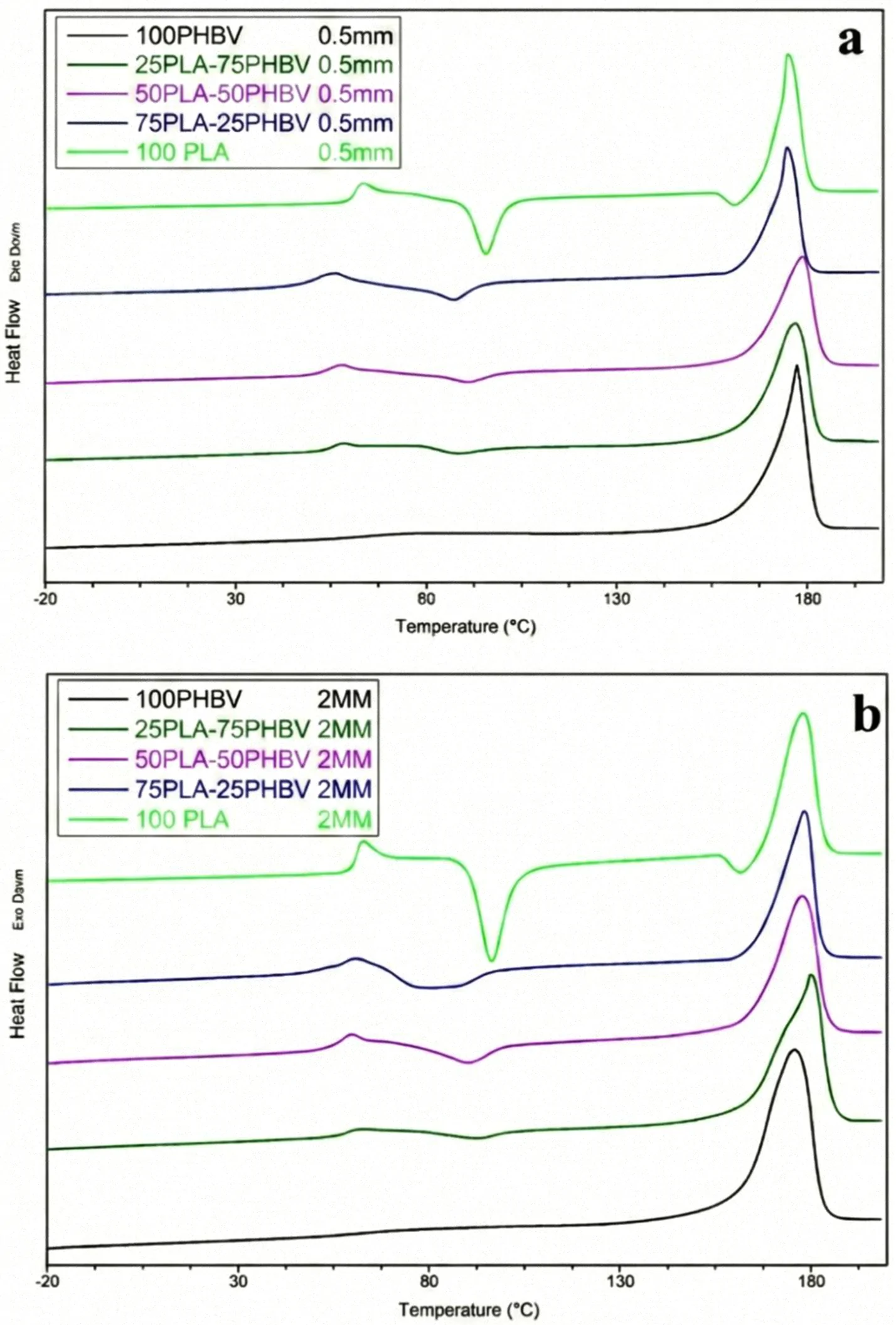
DSC thermograms of the PLA/PHBV blends with a) 0.5 mm thickness, b) 2 mm thickness.

Upon further heating, a pronounced cold crystallization peak (*T*_cc_) appears between 80 and 120 °C, indicating the reorganization of amorphous regions into crystalline domains. Prior to the main melting peak (*T*_m_), located at approximately 180 °C, a weak premelting endotherm is detected, which is commonly associated with molecular rearrangements within imperfect crystalline structures preceding complete fusion. In contrast, PHBV is characterized by an intrinsic glass transition temperature typically reported in the range of −5 to −10 °C;^40,41^ however, this transition was not clearly detected in the present thermograms. Instead, a subtle endothermic transition around 60 °C was observed and attributed to the rigid amorphous fraction (RAF) of PHBV.^42^

The RAF is a diffuse amorphous interphase (a region of finite thickness with graded properties) whose segmental mobility is constrained, typically near crystal lamellae due to chain anchoring (loops/tie molecules).

In composites, a similar rigid amorphous fraction can also form near filler surfaces or in highly entangled regions.^43^ Chains in this interphase experience mechanical and topological constraints, leading to a broadened and often shifted glass-transition response (longer relaxation times) because their dynamics are strongly restricted. PHBV exhibits a sharp melting peak between 160 and 180 °C, reflecting the fusion of highly ordered crystalline domains. Due to the significant overlap between the melting peaks of PLA and PHBV, it was not possible to accurately decouple and determine the exact crystallinity degrees of the individual components within the blends. Consequently, quantitative crystallinity was calculated only for the neat polymers, and the thermal behavior of the intermediate blends is interpreted qualitatively based on expected phase contributions. For the neat PLA, crystallinity values of 25.7% and 19.3% were obtained for specimens with thicknesses of 0.5 mm and 2 mm, respectively. In contrast, PHBV had substantially higher crystallinity, reaching approximately 66% for both thicknesses, confirming its markedly faster crystallization kinetics compared to PLA. According to the literature, the larger crystallization ability of PHBV is related to its more flexible molecular structure. The presence of hydroxyvalerate (HV) units disrupts chain regularity, reduces the energy barrier of crystallization, and facilitates nucleation and crystal growth.^44^ Moreover, the lower glass transition temperature of PHBV compared to PLA leads to an increased chain mobility, further promoting crystallization^52,53^ even if the difference in values measured in this study is negligible. The large crystallinity achieved during injection molding results in a limited mobile amorphous fraction in PHBV, with a significant portion of the amorphous phase behaving as a rigid amorphous fraction. This structural feature accounts for the relatively high apparent *T*_g_ associated with the RAF and contributes to the pronounced thermo-mechanical stability of PHBV at temperatures relevant to industrial composting.

In the PLA/PHBV blends, the incorporation of PHBV into PLA leads to an initial decrease in the glass transition temperature, which reaches a minimum before increasing with higher PHBV contents. This trend is also observed for specimens with thicknesses of 0.5 and 2 mm. Additionally, the cold crystallization peak of PLA is significantly reduced or completely suppressed, indicating restricted chain mobility and altered crystallization behavior in the blended systems. The melting region of the blends is characterized by multiple overlapping endothermic transitions, suggesting the existence of two distinct crystalline populations and the interaction of the amorphous phases.

No significant differences were observed between samples with different thicknesses, indicating that under the processing conditions adopted in this study, specimen thickness does not strongly affect crystallinity development or the subsequent disintegration behavior.

### 3.4 Dynamic Mechanical Analysis

The dynamic mechanical behavior of neat polymers and PLA/PHBV blends was investigated by DMA. The corresponding storage modulus (E′) and loss factor (tan δ) curves as a function of temperature are reported in Figure 6.

**Figure 6:**
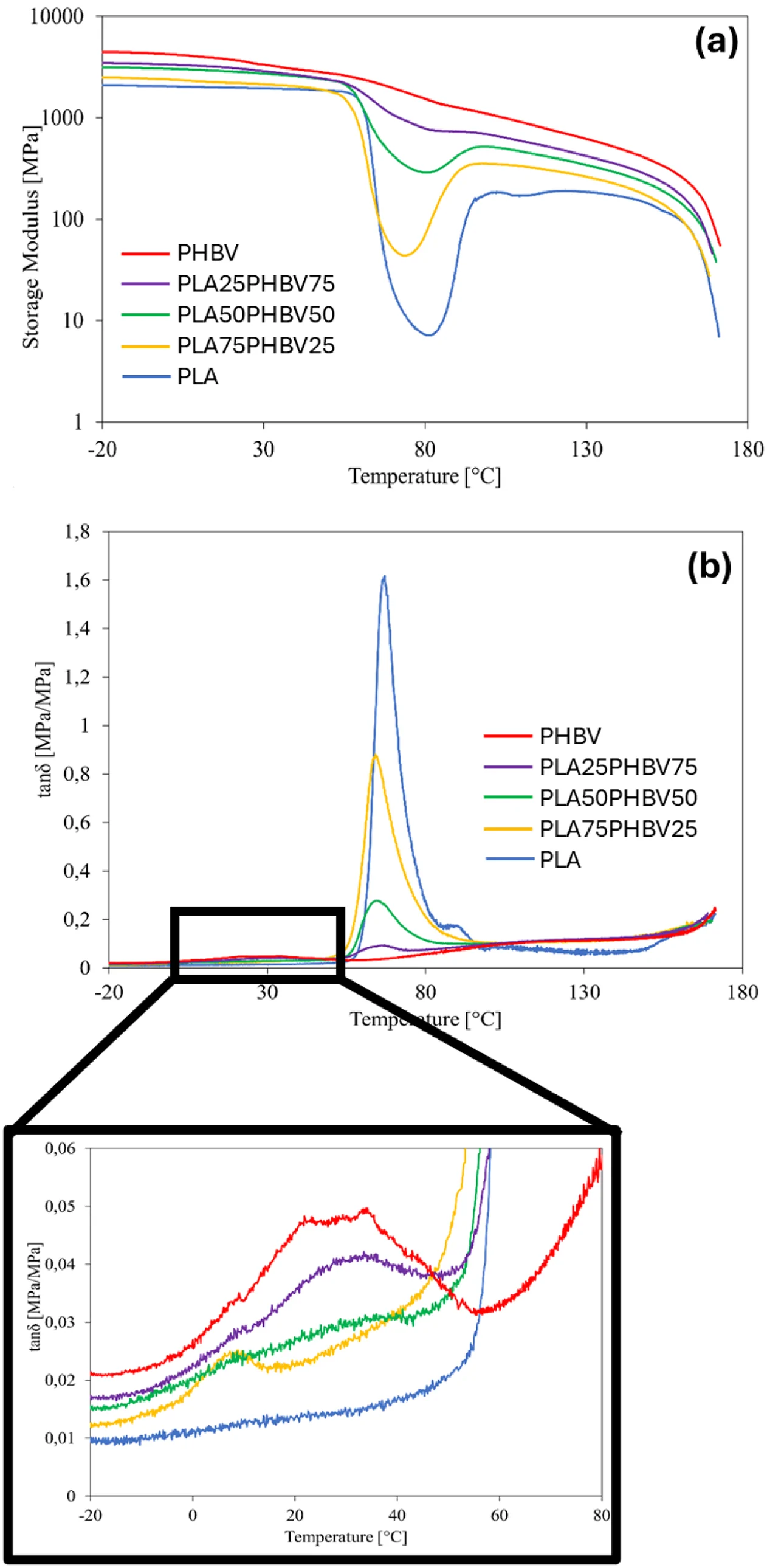
Temperature dependence of (a) storage modulus (E′) and (b) loss factor (tan δ) for neat PLA, neat PHBV, and the PLA/PHBV blends. The inset in (b) highlights the tan δ peak associated with the amorphous fraction of PHBV.

The distinct thermo-mechanical responses of neat PLA and neat PHBV are clearly observed (Figure 6a); at low and ambient temperatures, PLA exhibits a glassy response, characterized by a high storage modulus due to the restricted mobility of polymer chains below the glass transition temperature. Upon heating, a sharp decrease in E′ is detected as the temperature approaches *T*_g_, accompanied by a pronounced tan δ peak associated with the glass transition. Immediately after this transition, an increase in storage modulus is observed, which is attributed to cold crystallization of PLA,^45^ leading to a partial recovery of stiffness. At higher temperatures, a rubbery plateau is reached, followed by a rapid drop in E′ close to the melting temperature. In contrast, PHBV displays a significantly higher stiffness than PLA over the entire investigated temperature range. Its storage modulus decreases more gradually with increasing temperature, showing a nearly linear trend. A modest reduction in E′ around 30 °C, together with a small tan δ peak (inset of Figure 6b), can be ascribed to the glass transition of the amorphous fraction of PHBV.^46^

The thermo-mechanical responses of the PLA/PHBV blends fall between those of the neat components. In particular, the glass transition temperature associated with the PLA-rich phase progressively shifts toward that of PHBV as its content increases. Storage modulus values and tan δ peak temperatures for all compositions are summarized in Table 5. The *T*_g_ values determined by DMA are close to those obtained by DSC, although small differences are observed, which can be ascribed to the different experimental conditions (velocity of heat ramp, frequency, mass and shape of the samples, test operation principle) of the two techniques.

**Table 5:** Dynamic Mechanical Analysis (DMA) Results for PLA, PHBV, and PLA/PHBV Blends^a^

	E′ @ −20 °C [MPa]	E′ @ 20 °C [MPa]	E′ @ 40 °C [MPa]	T_g_ [°C]	PHBV, RAF related transition [°C]
**PLA**	2088 ± 1	1976 ± 2	1903 ± 1	67 ± 2	
**PLA75PHBV25**	2495 ± 1	2227 ± 3	2071 ± 4	66 ± 1	
**PLA50PHBV50**	3144 ± 1	2875 ± 3	2592 ± 5	65 ± 1	
**PLA25PHBV75**	3457 ± 1	3117 ± 6	2666 ± 7	66 ± 1	
**PHBV**	4438 ± 1	3717 ± 10	2997 ± 10		30 ± 2

a Storage modulus values (*E*′) at selected temperatures and glass transition temperatures (*T*_*g*_) determined from tan δ peaks are reported. Standard deviations are given in parentheses.

### 3.5 Disintegration Behavior under Laboratory Composting Conditions

The results of the laboratory-scale disintegration tests reveal distinct degradation behaviors for the neat polymers and the PLA/PHBV blends. Changes in the optical images of specimens with a thickness of 0.5 mm during composting are shown in Figure 7 and the thickness of 2 mm in Figure 8.

**Figure 7:**
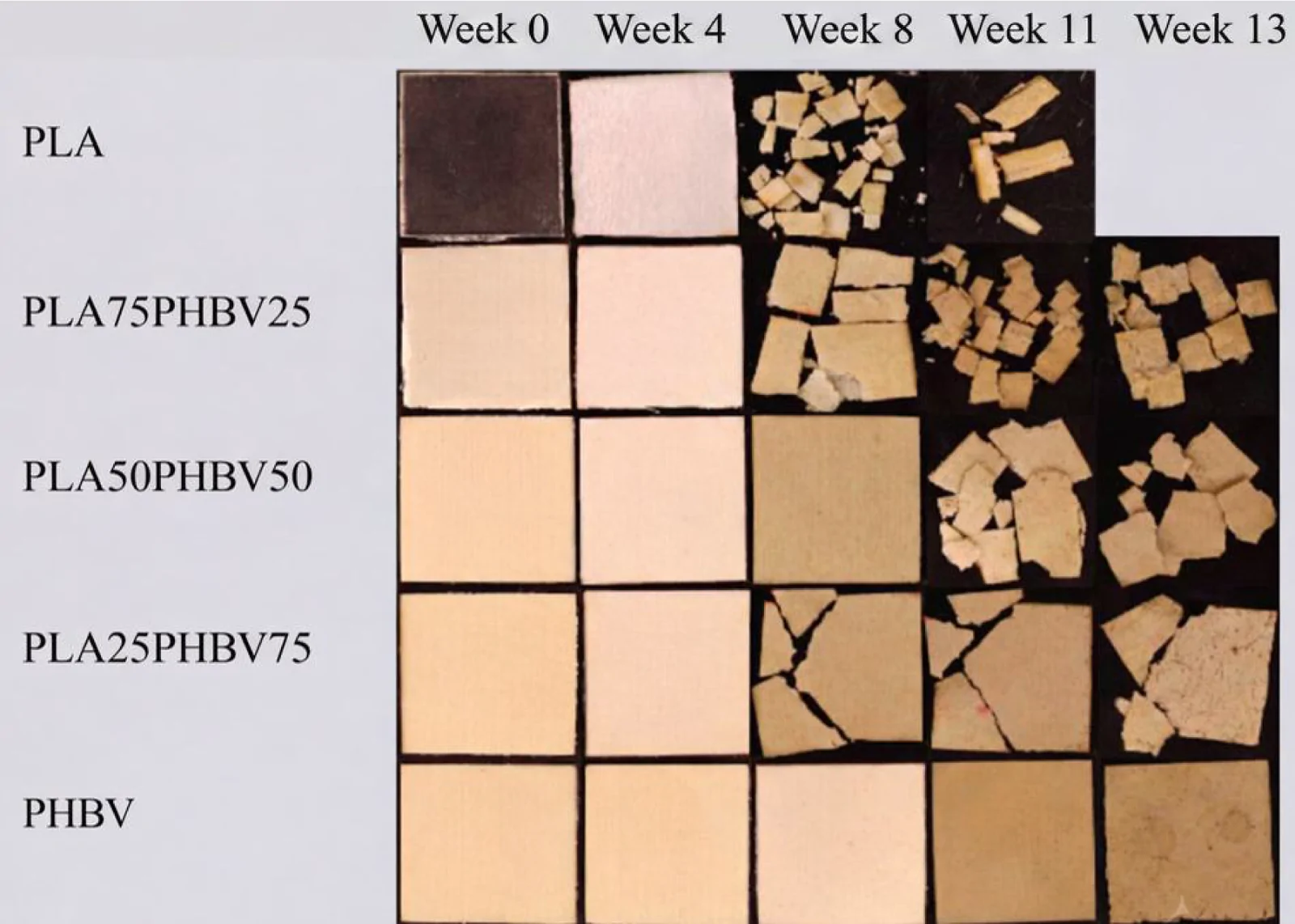
Optical images showing the disintegration behavior of PLA, PHBV, and PLA/PHBV blends with the specimen thickness of 0.5 mm under laboratory composting conditions.

**Figure 8:**
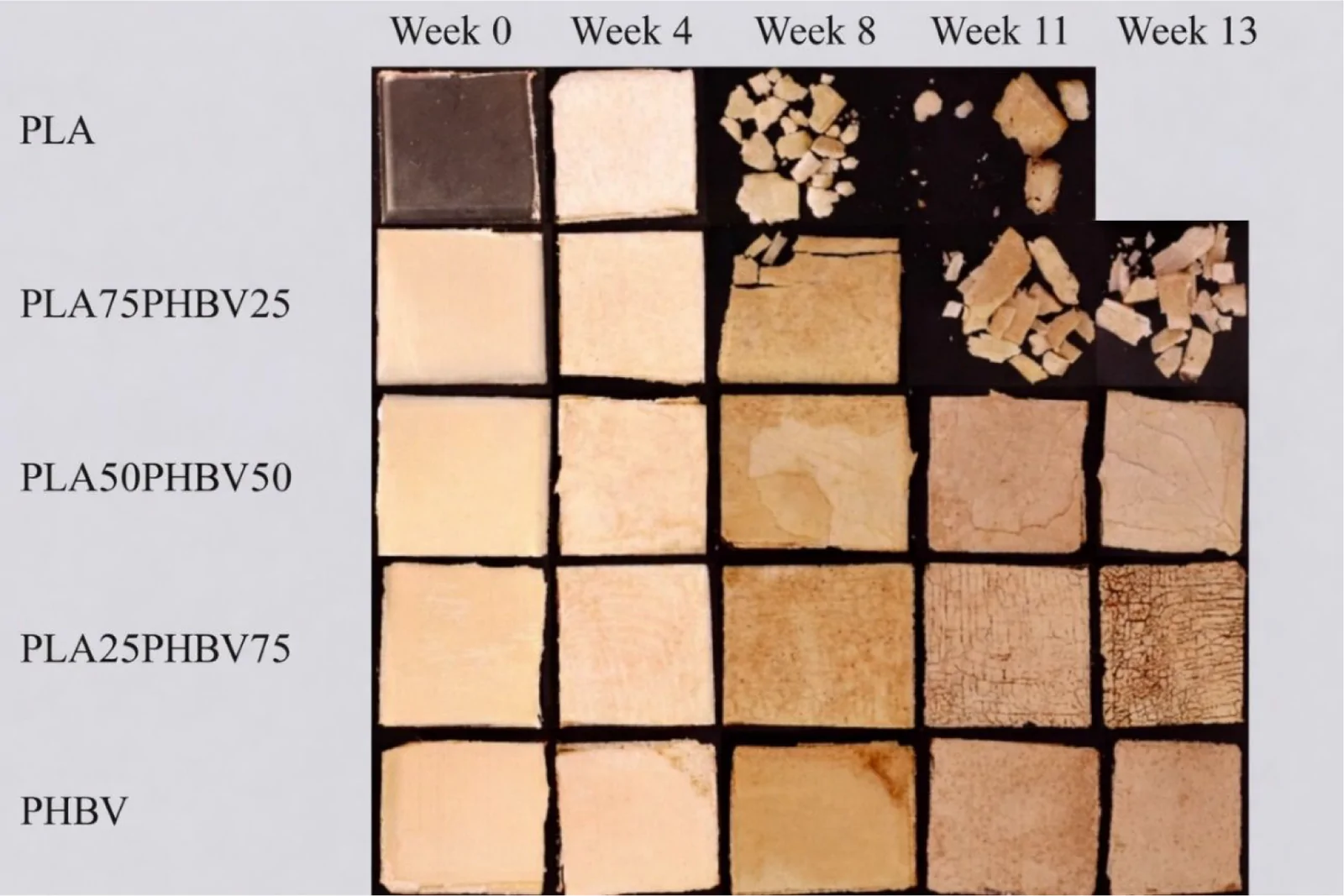
Optical images showing the disintegration behavior of PLA, PHBV, and PLA/PHBV blends with the specimen thickness of 2 mm under laboratory composting conditions.

Neat PLA exhibits complete disintegration within the test duration, regardless of specimen thickness, and shows the largest degree of disintegration among all investigated compositions. This behavior can be attributed to the considerable susceptibility of PLA to hydrolytic degradation under composting conditions, which leads to rapid molecular weight reduction and loss of structural integrity.

In contrast, neat PHBV displays the smallest degree of disintegration, with degradation strongly dependent on specimen thickness. Thicker PHBV samples show limited mass loss, whereas thinner specimens exhibit a slightly larger degree of disintegration. This thickness dependence is intrinsically linked to the surface-area-to-volume ratio. Because the experimental design maintains a constant test material-to-compost mass ratio, thinner specimens inevitably expose a larger surface area to the compost medium. Consequently, the effects of sample thickness and exposed surface area are coupled, both dictating the rate of surface-driven disintegration. Although industrial composting temperatures around 58 °C are generally considered favorable for the disintegration of bioplastics such as PHBV, due to enhanced activity of thermophilic microorganisms,^47^ effective disintegration requires not only elevated temperature but also appropriate microbial diversity and enzyme availability. The present results suggest that the large crystallinity of PHBV, together with the significant presence of a rigid amorphous fraction (RAF), strongly inhibits degradation. Restricted molecular mobility within these constrained amorphous regions likely hampers enzyme–substrate interactions and limits the fraction of chains that can rearrange to reach an active conformation, so that degradation remains mainly superficial and proceeds slowly. Alternatively, the specific enzymes required for efficient PHBV degradation may not have been present or sufficiently active under the tested composting conditions, further contributing to the observed resistance to disintegration.

### 3.6 Effect of Blend Composition on Disintegration Kinetics

As shown by Figures 9 and 10, the degree of disintegration increases with increasing PLA content for the PLA/PHBV blends. The disintegration curves indicate an approximately linear trend at low PLA concentrations, whereas kinetics of degradation is different in PLA-rich compositions.

**Figure 9:**
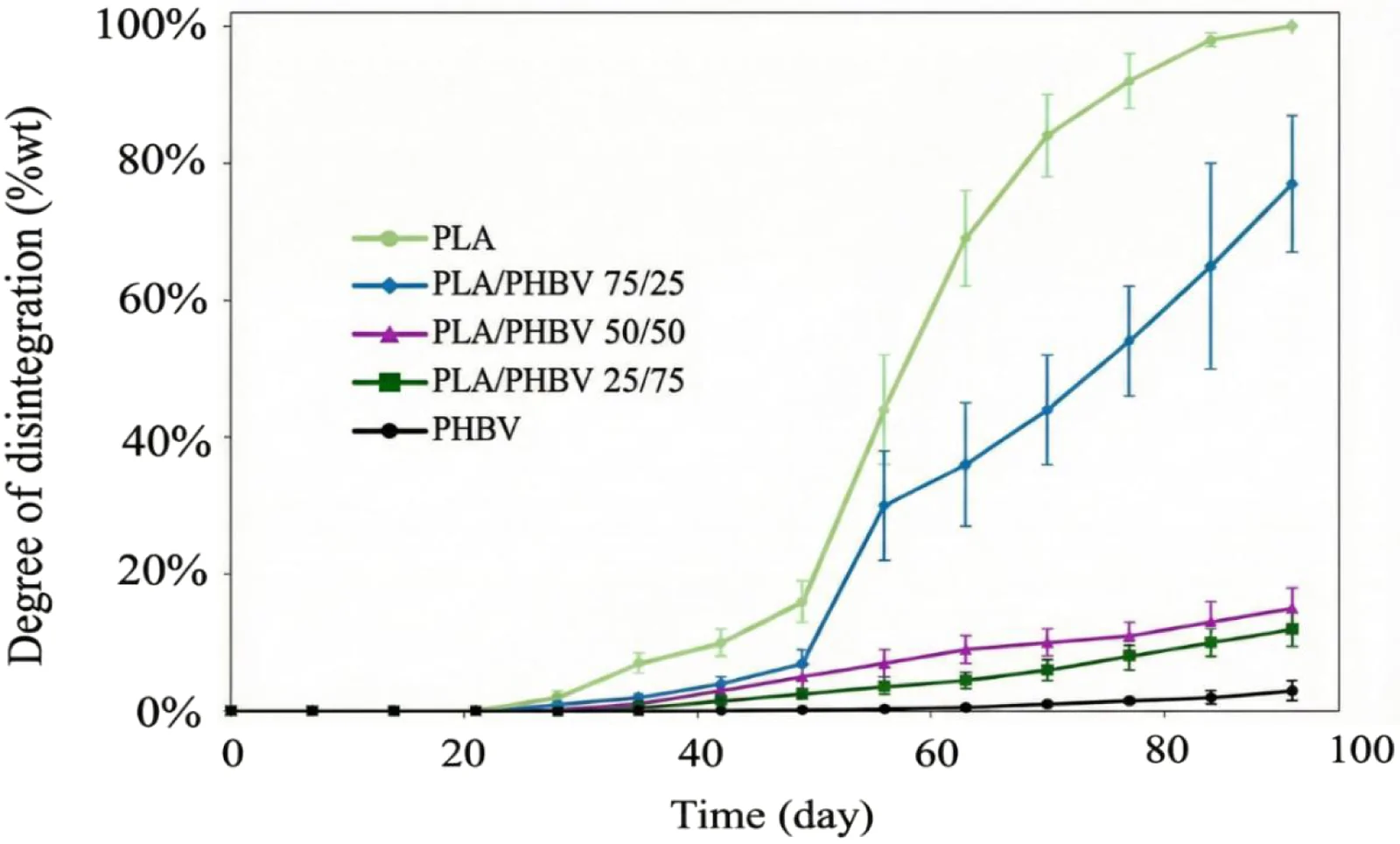
Degree of disintegration as a function of composting time for PLA/PHBV specimens with a thickness of 0.5 mm.

**Figure 10:**
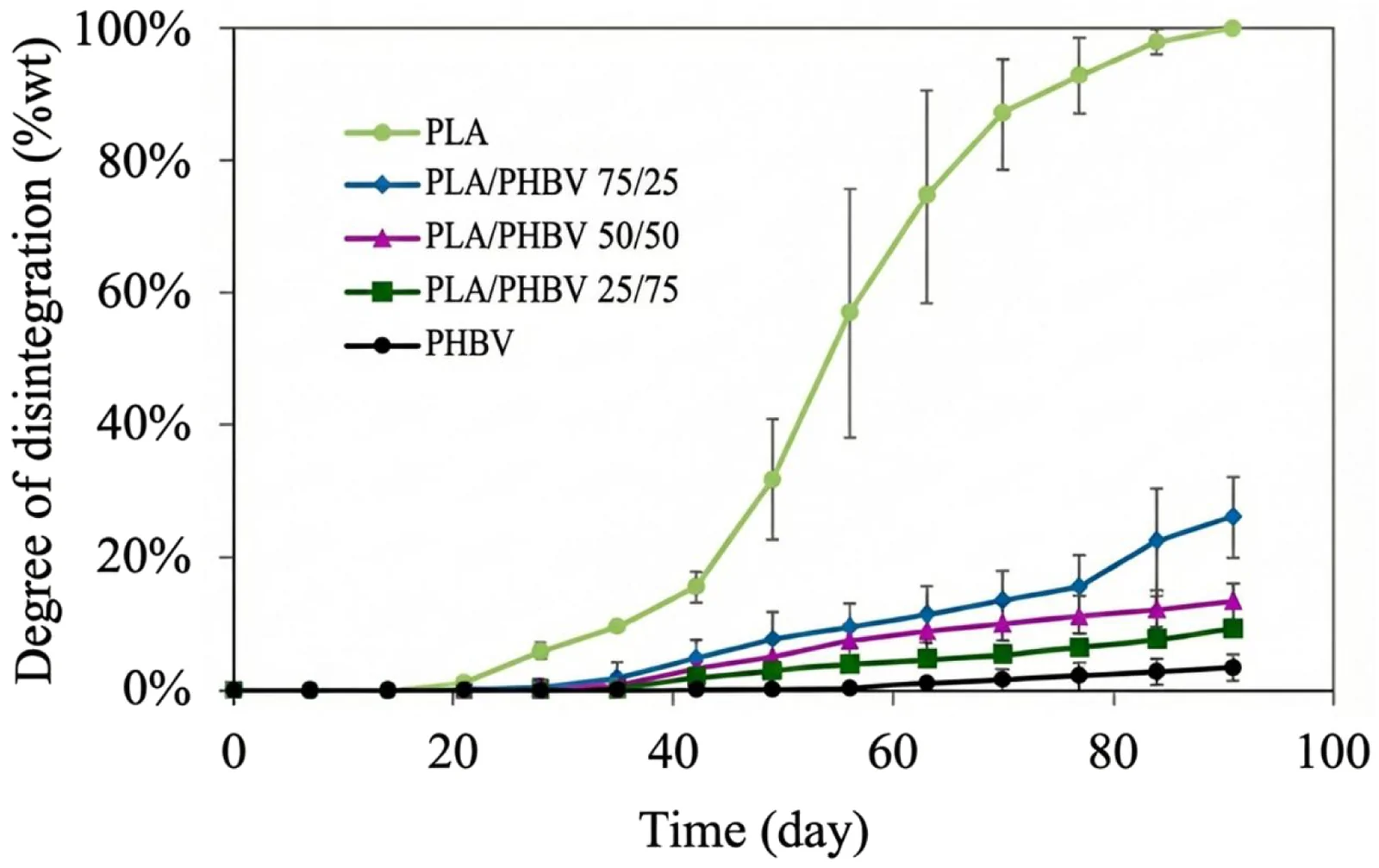
Degree of disintegration as a function of composting time for PLA/PHBV specimens (thickness of 2 mm).

In blends containing larger fractions of PLA, disintegration accelerates markedly. While PLA is known to be susceptible to hydrolytic degradation under composting conditions, the degradation of these aliphatic polyesters is mainly driven by enzymatic action. The absence of disintegration during the first 20 days represents a lag phase primarily associated with water absorption, microbial colonization, and the initial action of extracellular enzymes. During this stage, microorganisms secrete depolymerases that adsorb onto the polymer surface and catalyze the cleavage of ester bonds into soluble oligomers and monomers, which are subsequently assimilated.^48^ The pronounced difference in the disintegration rates between the 0.5 mm and 2 mm specimens further highlights the surface-dependent nature of this enzymatic degradation. By maintaining a constant test material-to-compost mass ratio, the thinner specimens intrinsically exposed a significantly larger surface area to the medium, thereby facilitating faster enzyme adsorption and surface erosion.

### 3.7 FTIR Analysis of Disintegrated Samples

To further elucidate the degradation mechanisms, Fourier transform infrared (FTIR) spectroscopy (Figure 11) was employed to analyze samples before the starting of disintegration tests and after the end of the test (day 100). For neat PLA, a pronounced decrease in the intensity of the ester-related absorption band is observed at approximately 1084 cm^–1^ after composting, indicating extensive chain scission during degradation. This result is consistent with the complete disintegration of PLA.^49^

**Figure 11:**
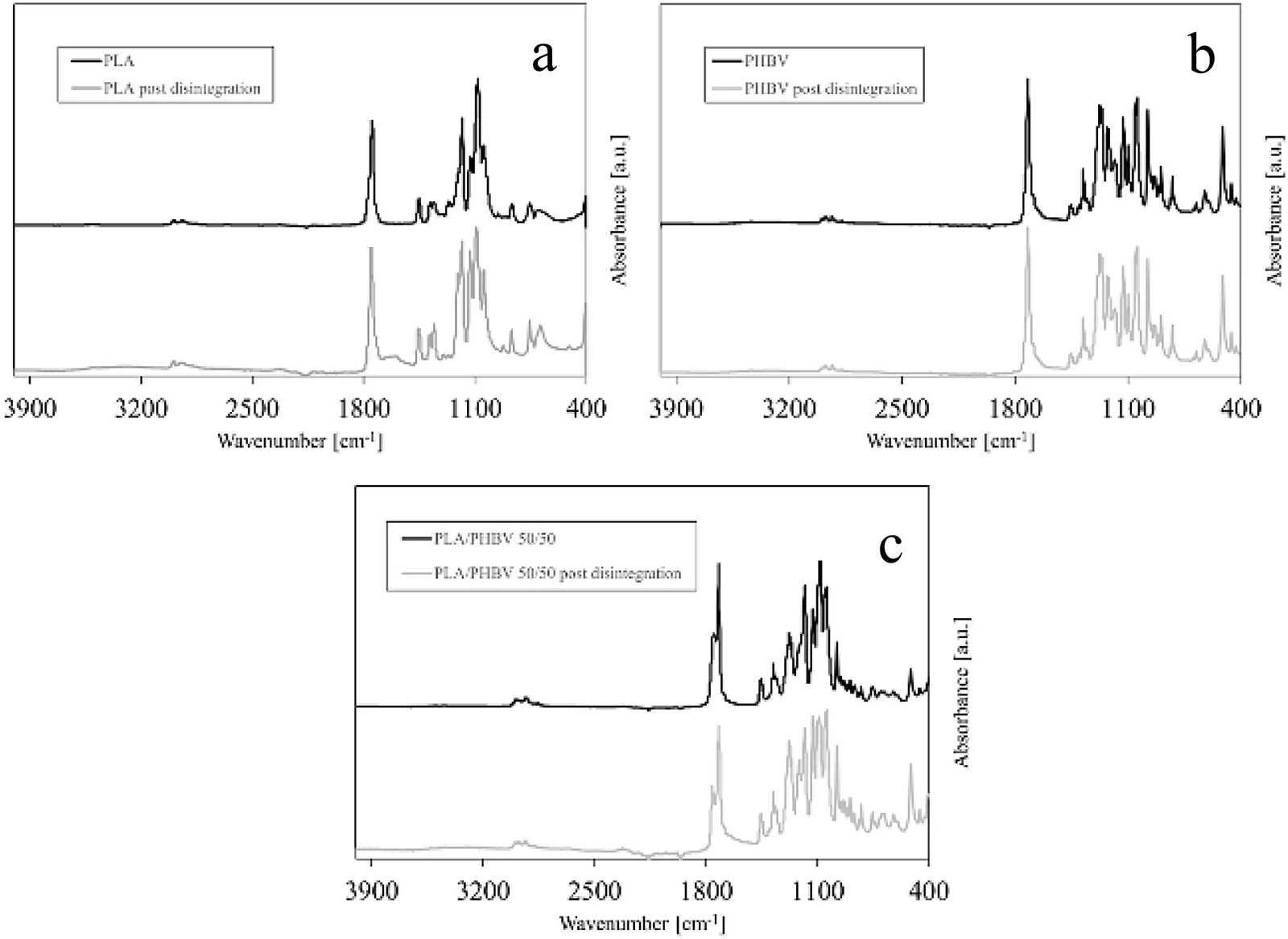
ATR–FTIR spectra of samples before and after laboratory composting: (a) neat PLA, (b) neat PHBV, and (c) PLA/PHBV 50/50 blend as a representative composition.

In contrast, no significant changes are detected in the ATR-FTIR spectrum of neat PHBV before and after disintegration tests. While ATR-FTIR exclusively probes surface chemistry and cannot quantify bulk molecular weight reduction, this lack of spectral alteration suggests a strong surface resistance of PHBV to physical fragmentation under the tested conditions, consistent with its high crystallinity.

The FTIR spectra of PLA/PHBV blends exhibit characteristic carbonyl absorption bands at approximately 1760 cm^–1^ for PLA and 1720 cm^–1^ for PHBV. After disintegration, a relative decrease in the intensity of the PLA carbonyl band is observed, whereas the PHBV-related band remains largely unchanged. This preferential reduction becomes more pronounced in PLA-rich blends and reflects the selective degradation of the PLA phase. These spectroscopic results corroborate the disintegration data and further confirm that the degradation of the blends is primarily dictated by the PLA content.

### 3.8 SEM Analysis of Surface Morphology after Disintegration

Figure 12 presents SEM micrographs of the surfaces of PLA, PHBV, and PLA/PHBV blends before and after the laboratory disintegration tests.

**Figure 12:**
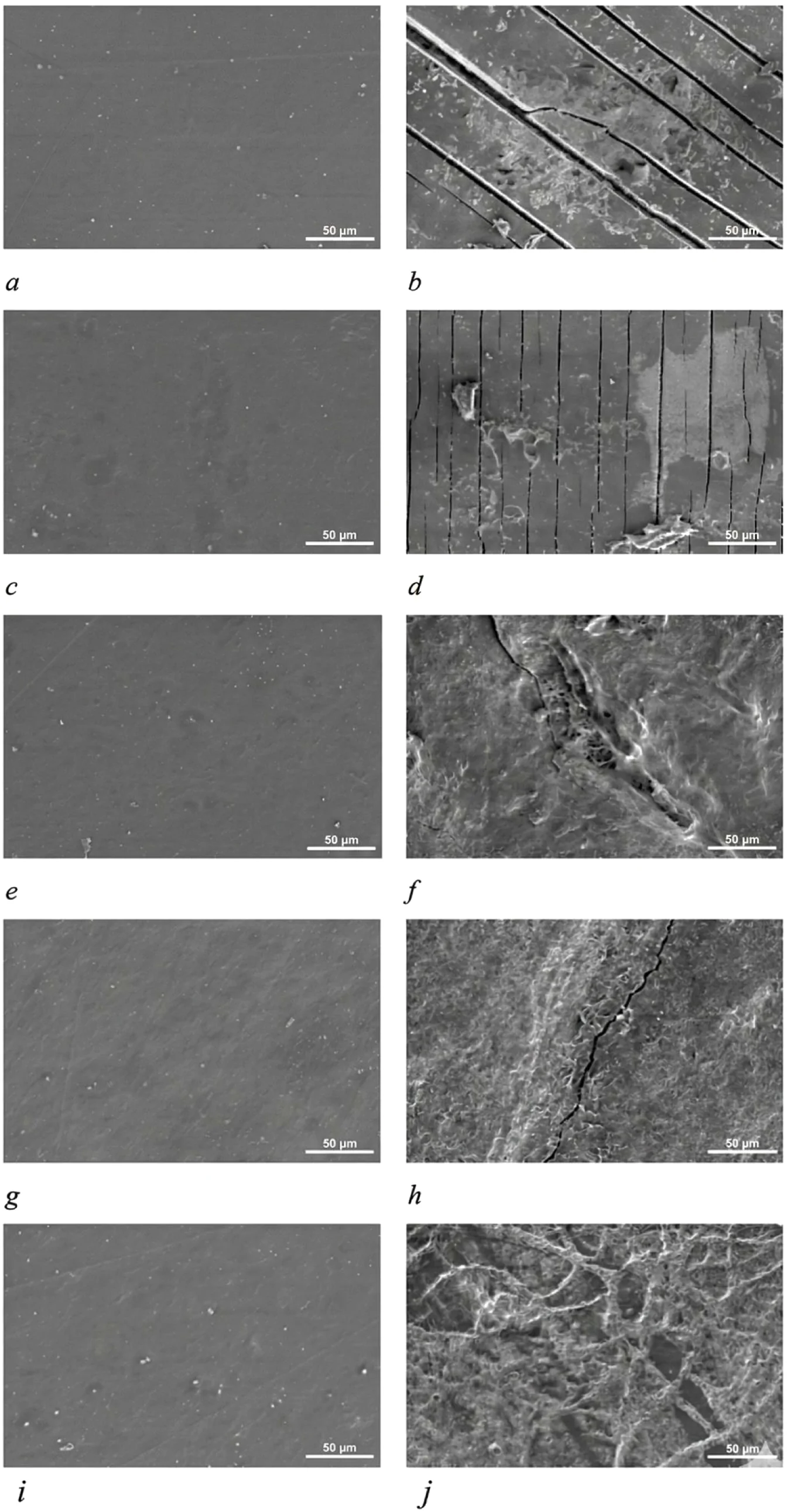
SEM images (1000×) of samples, before (on the left) and after (on the right) disintegration tests, respectively: PLA (a, b), PLA/PHBV 75/25 (c, d), PLA/PHBV 50/50 (e, f), PLA/PHBV 25/75 (g, h), PHBV (i, j).

Prior to composting, all samples exhibit smooth and homogeneous surfaces. After composting, SEM micrographs of PLA-rich samples reveal parallel crack-like features. Their preferential alignment suggests the release of residual molding stresses and structural relaxation. These phenomena are particularly relevant for PLA, as the composting temperature is close to its glass transition, promoting embrittlement. Such surface damage increases the effective surface area and exposes the polymer at the crack walls. Since both PLA and PHBV exhibit very low water uptake (typically below 0.5%),^50,51^ the disintegration process is not governed by water diffusion into the bulk. Instead, degradation is primarily a surface-level phenomenon.^52^ This is further supported by the fact that the enzymes responsible for catalyzing the hydrolytic chain scission are large molecules that cannot penetrate the intact polymer matrix; consequently, biological activity occurs exclusively on the exposed surfaces (the outer surface and the newly formed crack walls).^53^ The parallel cracks observed in the PLA-based matrices therefore promote degradation mainly by accelerating physical fragmentation and increasing the surface area available for enzymatic attack. In the case of PLA, the increased molecular mobility near its glass transition facilitates the interaction between the polymer chains and the active sites of the enzymes. In contrast, physical aging effects are negligible for PHBV, owing to its high crystallinity and a *T*_g_ significantly below the composting temperature. PHBV surfaces exhibit only minor modifications, associated with localized microbial colonization and general hydrolytic or thermo-oxidative aging, without the extensive fracturing observed in the PLA phase. This behavior is consistent with the large crystallinity of PHBV, as enzymatic degradation preferentially initiates in amorphous regions, while crystalline domains are largely resistant to attack.^54^ The PLA/PHBV blends exhibit intermediate surface morphologies, with increasing surface degradation observed as the PLA content increases. These results are consistent with both disintegration kinetics and FTIR analysis and confirm the dominant role of PLA in governing the disintegration of the blends.

### 3.9 Disintegration Behavior under Pilot-Scale Composting Conditions

To verify the trends observed in laboratory-scale tests under more realistic industrial composting conditions, pilot-scale disintegration tests were conducted on PHBV, which exhibited the lowest disintegration degree among all investigated materials. The analysis focused exclusively on PHBV, as it represents the most critical case in terms of compostability.

Throughout the test, compost maturity was verified, oxygen concentration in the exhaust air remained above 10%, and pH values were consistently larger than 5, fully satisfying the requirements of ISO 16929:2021. Despite these favorable composting conditions, both type of samples exhibited very limited disintegration. After 12 weeks, the mass loss was approximately 5% for both type of sample, indicating that aging did not significantly influence the disintegration behavior of PHBV under the tested conditions. These results (Figure 13) confirm that PHBV shows a remarkably small degree of disintegration even under controlled pilot-scale composting conditions that are compliant with industrial standards.

**Figure 13:**
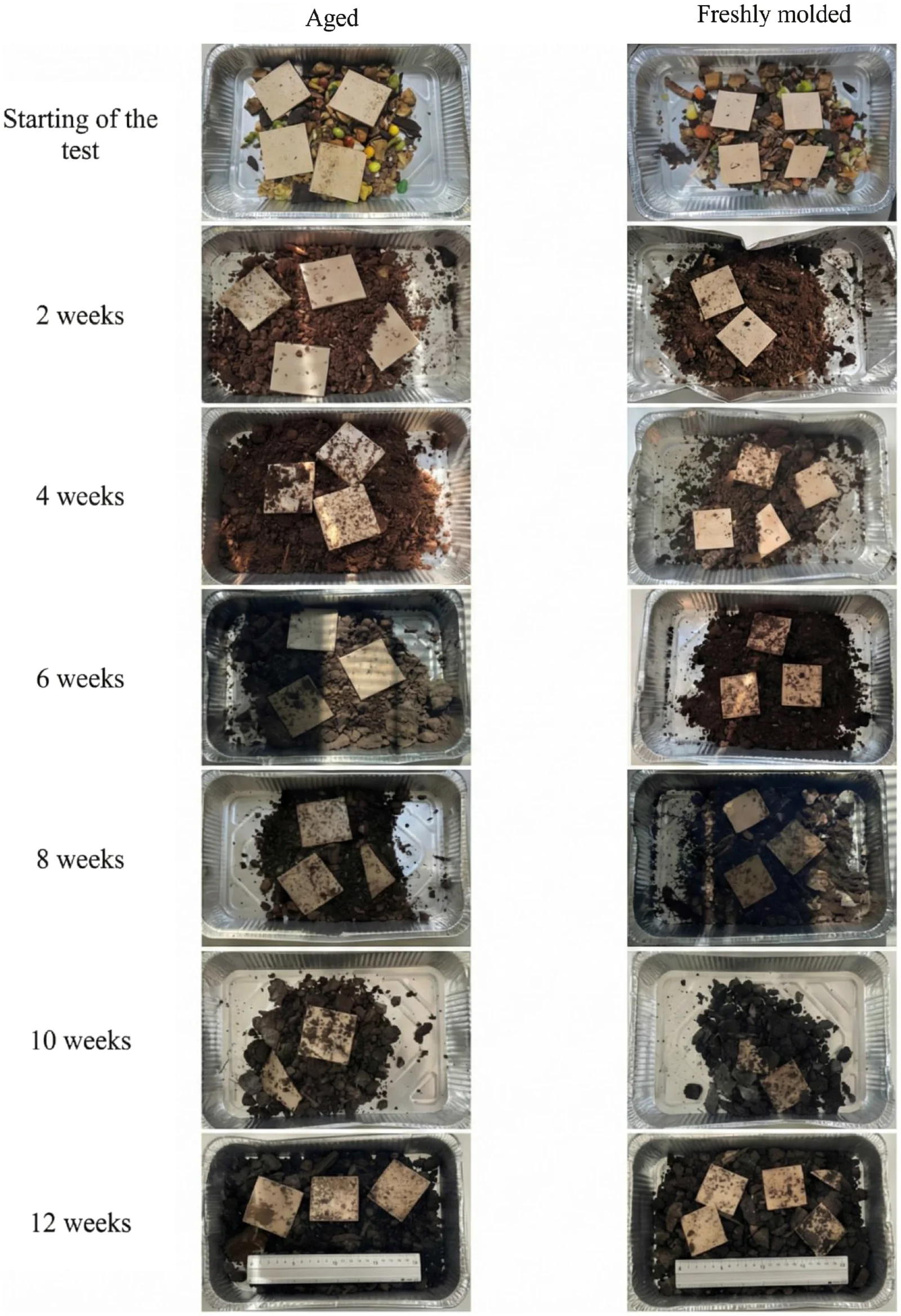
Representative images of PHBV specimens during pilot-scale composting tests performed according to the ISO 16929:2021 standard. Both freshly injection-molded and six-month-aged samples exhibit minimal disintegration, with a mass loss of approximately 5% after 12 weeks under controlled industrial composting conditions.

The limited degradation observed can be attributed to the inherently high crystallinity of PHBV and to the presence of a substantial rigid amorphous fraction. These structural features reduce molecular mobility and limit enzyme–substrate interactions, resulting in a slow, surface-controlled disintegration process, even under pilot-scale composting conditions.

The pilot-scale findings are fully consistent with laboratory-scale results and further emphasize the critical role of polymer structure and morphology in governing compostability. In particular, the data demonstrate that, in the absence of specifically tailored enzymatic systems, PHBV may fail to meet the disintegration criteria defined in EN 13432, despite being widely classified as a biodegradable polymer. This emphasizes the importance of experimentally verifying compostability under realistic conditions rather than relying solely on material classification or chemical composition.

It is worth noting that the PHBV grade investigated in this study is commercially certified as OK Compost Industrial. The limited disintegration observed for injection-molded specimens does not contradict this certification but rather emphasizes the strong influence of processing history on compostability. It is plausible that certification tests were performed on samples subjected to different thermo-mechanical conditions, potentially resulting in smaller crystallinity and a larger mobile amorphous fraction. In contrast, the injection molding conditions adopted in the present work promoted large crystallinity and the formation of a significant rigid amorphous fraction, considerably reducing molecular mobility and disintegration under composting conditions.

## 4 Conclusions

This study demonstrates that the biodegradability and disintegration behavior of PLA/PHBV blends cannot be assumed a priori based solely on their nominal chemical composition. Instead, the experimental results clearly show that disintegration is governed by a complex interplay among processing history, crystallinity, phase morphology, and the specific conditions of the composting environment.

The systematic investigations conducted across the full composition range reveal that PHBV, despite being widely classified as a biodegradable polymer, exhibits a remarkably small degree of disintegration under both laboratory-scale and pilot-scale composting conditions. This behavior is primarily attributed to its high crystallinity and to the presence of a substantial rigid amorphous fraction, which together impart elevated thermo-mechanical stability and strongly reduce molecular mobility and enzymatic accessibility at temperatures typical for industrial composting processes. Consistent with this interpretation, the limited disintegration observed for injection-molded PHBV specimens despite the material being commercially certified as OK Compost Industrial, further underscores the critical role of processing-induced structural features in determining effective disintegration.

In PLA/PHBV blends, the degree of disintegration increases progressively with increasing PLA content, indicating that PLA governs the overall degradation kinetics of the blends. Neither blend composition nor specimen thickness was found to overcome significantly the inhibitory effect of PHBV crystallinity on disintegration under the investigated conditions. These findings prove that the presence of a biodegradable component within a polymer blend does not necessarily guarantee effective degradation of the entire material.

Importantly, the agreement between laboratory-scale and pilot-scale results underscores the necessity of validating disintegration performance under realistic and standardized industrial conditions. The present data demonstrate that, in the absence of specifically tailored enzymatic activity, PHBV may fail to meet the disintegration criteria required by standards such as EN 13432, despite favorable composting environments and compliance with ISO testing protocols.

Overall, this work emphasizes the importance of adopting an integrated evaluation strategy that correlates processing-induced structural features, thermal and mechanical stability, and environmental degradation conditions. Such an approach is essential for the reliable assessment and rational design of biobased and biodegradable polymeric materials intended for compostable applications, ensuring that material classification aligns with actual end-of-life performance.

## Data Availability

The data underlying this study are not publicly available because they constitute a partial data set of an ongoing, larger research project and contain unpublished raw data intended for future studies. The data are available from the corresponding author upon reasonable request.
